# Reduced fatty acid uptake aggravates cardiac contractile dysfunction in streptozotocin-induced diabetic cardiomyopathy

**DOI:** 10.1038/s41598-020-77895-1

**Published:** 2020-11-30

**Authors:** Yogi Umbarawan, Ryo Kawakami, Mas Rizky A. A. Syamsunarno, Norimichi Koitabashi, Hideru Obinata, Aiko Yamaguchi, Hirofumi Hanaoka, Takako Hishiki, Noriyo Hayakawa, Hiroaki Sunaga, Hiroki Matsui, Masahiko Kurabayashi, Tatsuya Iso

**Affiliations:** 1 Department of Cardiovascular Medicine, Gunma University Graduate School of Medicine, 3-39-22 Showa-machi, Maebashi, Gunma 371-8511 Japan; 2 Education and Research Support Center, Gunma University Graduate School of Medicine, 3-39-22 Showa-machi, Maebashi, Gunma 371-8511 Japan; 3 Department of Bioimaging Information Analysis, Gunma University Graduate School of Medicine, 3-39-22 Showa-machi, Maebashi, Gunma 371-8511 Japan; 4 Department of Laboratory Sciences, Gunma University Graduate School of Health Sciences, 3-39-22 Showa-machi, Maebashi, Gunma 371-8511 Japan; 5 Department of Biochemistry, Keio University School of Medicine, 35 Shinano-machi, Shinjuku-ku, Tokyo, 160-8582 Japan; 6 Clinical and Translational Research Center, Keio University School of Medicine, 35 Shinano-machi, Shinjuku-ku, Tokyo, 160-8582 Japan; 7 Department of Internal Medicine, Faculty of Medicine Universitas Indonesia, Jl. Salemba Raya no. 6, Jakarta, 10430 Indonesia; 8 Department of Biochemistry and Molecular Biology, Universitas Padjadjaran, Jl. Raya Bandung Sumedang KM 21, Jatinangor, West Java 45363 Indonesia; 9 Center for Liberal Arts and Sciences, Ashikaga University, 268-1 Omae-machi, Ashikaga, Tochigi 326-8558 Japan

**Keywords:** Diabetes, Cardiovascular biology, Metabolomics, Metabolism

## Abstract

Diabetes is an independent risk factor for the development of heart failure. Increased fatty acid (FA) uptake and deranged utilization leads to reduced cardiac efficiency and accumulation of cardiotoxic lipids, which is suggested to facilitate diabetic cardiomyopathy. We studied whether reduced FA uptake in the heart is protective against streptozotocin (STZ)-induced diabetic cardiomyopathy by using mice doubly deficient in fatty acid binding protein 4 (FABP4) and FABP5 (DKO mice). Cardiac contractile dysfunction was aggravated 8 weeks after STZ treatment in DKO mice. Although compensatory glucose uptake was not reduced in DKO-STZ hearts, total energy supply, estimated by the pool size in the TCA cycle, was significantly reduced. Tracer analysis with ^13^C_6_-glucose revealed that accelerated glycolysis in DKO hearts was strongly suppressed by STZ treatment. Levels of ceramides, cardiotoxic lipids, were similarly elevated by STZ treatment. These findings suggest that a reduction in total energy supply by reduced FA uptake and suppressed glycolysis could account for exacerbated contractile dysfunction in DKO-STZ hearts. Thus, enhanced FA uptake in diabetic hearts seems to be a compensatory response to reduced energy supply from glucose, and therefore, limited FA use could be detrimental to cardiac contractile dysfunction due to energy insufficiency.

## Introduction

Diabetic cardiomyopathy occurs among patients with diabetes independently of other cardiovascular risk factors including hypertension and ischemic heart disease^1–3^. Although severity of diabetic cardiomyopathy progresses from subtle structural and functional abnormalities to end-stage heart failure, typical diabetic cardiomyopathy displays left ventricular diastolic and systolic dysfunction, left ventricular hypertrophy and interstitial fibrosis^4–8^. It has been proposed that diabetic cardiomyopathy is induced by multifactorial mechanisms including increased myocardial fatty acid (FA) utilization and lipid storage, sustained hyperglycemia, reduced glucose consumption, compromised mitochondrial energetics, impaired Ca^2+^ handling, increased connective tissue content, increased reactive oxygen species (ROS) production, and enhanced inflammatory responses^4–8^. Among these possibilities of causative burdens, lipotoxicity has been suggested to have a significant role in the development of diabetic cardiomyopathy.

Abnormal FA utilization in the heart has been shown in diabetes in humans and animals^4–11^. FA uptake and utilization are presumably accelerated to compensate for decreased glucose use, which is often accompanied by increased expression of genes associated with FA utilization, such as peroxisome proliferator-activated receptor α (PPARα) and its target genes^12–15^. In cardiomyocytes, excessive FA is converted into other lipid intermediates such as neutral triacylglycerol (TG) and others with lipotoxic effects (e.g. diacylglycerol [DAG] and ceramides). Increased levels of ceramides can stimulate inflammatory signaling and facilitate ROS production, which in turn results in impairment of mitochondrial energetics. Reduced cardiac efficiency (cardiac work/oxygen consumption) by deranged FA use is also suggested to be a causative burden for diabetic cardiomyopathy. To date, the lipotoxicity hypothesis is prevailing as a key event driving cardiac dysfunction in diabetes.

Fatty acid binding protein 4 (FABP4) and FABP5, intracellular lipid chaperons, are abundantly expressed in capillary endothelial cells in the heart and skeletal muscle, in addition to adipocytes and macrophages^16,17^. We recently reported that FABP4/5 in capillary endothelium facilitates FA transport from circulation into these tissues (known as trans-endothelial FA transport), particularly in the heart^16^. Importantly, in mice with the genetic deletion of both FABP4 and FABP5 (double knockout, DKO), cardiac FA uptake is reduced by 30% compared to wild-type (WT) while compensatory glucose uptake is 20-fold higher. We further found that a reduction in FA use with elevated glucose uptake renders DKO hearts more susceptible to cardiac dysfunction under increased workload by transverse aortic constriction^18^. Cardiac dysfunction in DKO-TAC mice is associated with a reduction in total energy supply relative to energy demand, which is partially rescued by supplementation of medium chain FA. These findings provided an important notion that long chain FA are pivotal fuels to generate sufficient energy for preserved cardiac contraction under increased workload and not toxic for pressure-overloaded hearts. We also demonstrated that FABP4/5 DKO hearts are a useful model to evaluate contribution of reduced FA utilization to pathophysiology of heart failure models^18,19^.

In this study, we tested the hypothesis whether reduced FA utilization in DKO mice is protective against diabetic cardiomyopathy generated by streptozotocin (STZ) treatment. Contrary to our expectation, we found that cardiac contractile dysfunction was exacerbated in DKO-STZ mice. Total energy supply, estimated by the pool size in the tricarboxylic acid (TCA) cycle, was reduced in DKO-STZ hearts, which could account for reduced contractile function in DKO-STZ hearts. Our data suggest that enhanced FA utilization in diabetic hearts occurs to compensate for reduced glucose use, and therefore, that limitation of FA utilization is not always beneficial for diabetic cardiomyopathy.

## Results

### Deterioration of left ventricular contractile dysfunction in STZ-treated DKO mice

**Figure 1:**
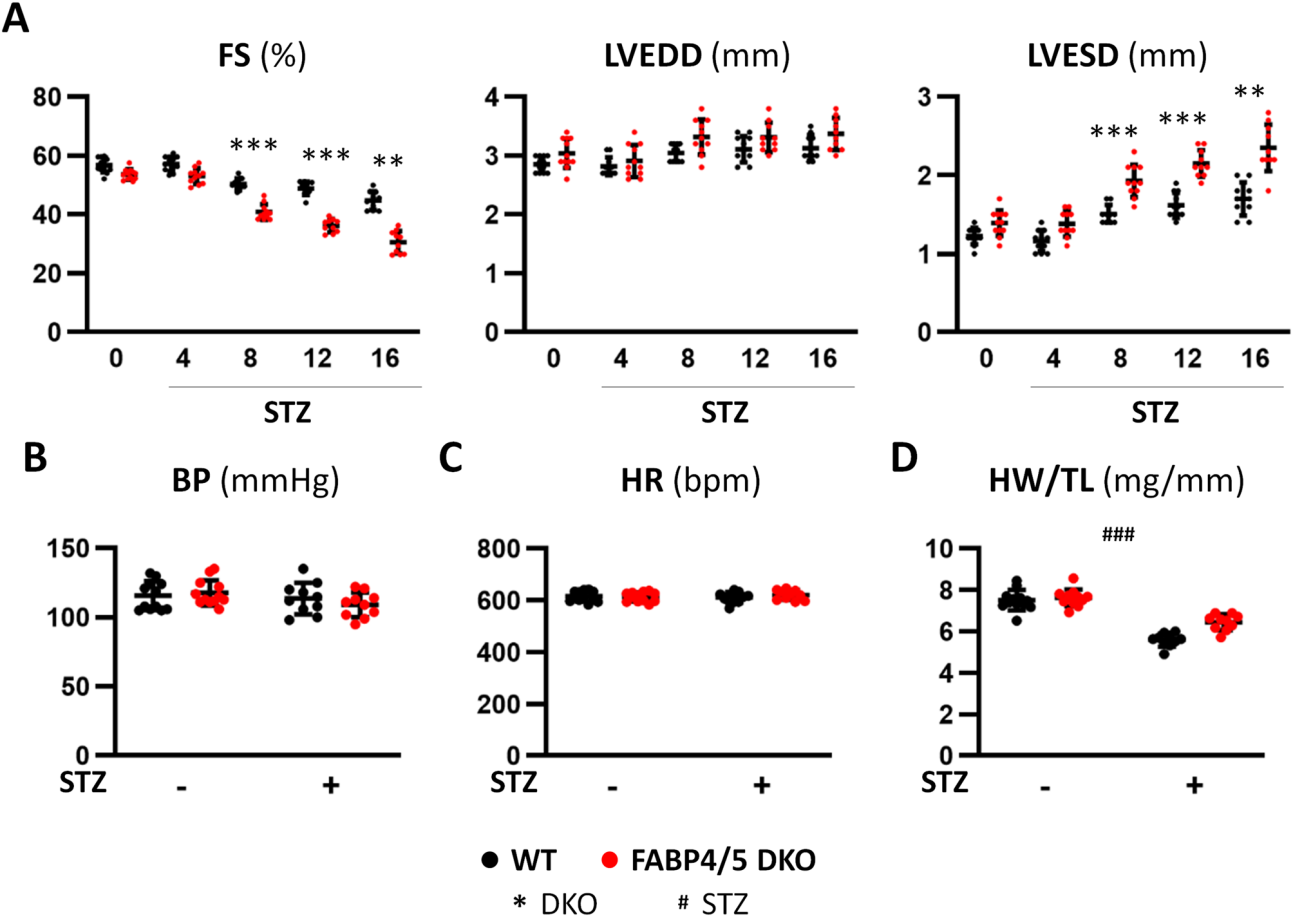
Cardiac contractile dysfunction was more deteriorated in DKO-STZ mice. (**A**) Cardiac function was estimated by echocardiography before and 4, 8, 12 and 16 weeks after STZ treatment (n = 10–12). *FS* fractional shortening, *LVEDD* left ventricular end-diastolic diameter, *LVESD* left ventricular end-systolic diameter. (**B**,**C**) Systolic blood pressure (BP) and heart rate (HR) were not significantly different. (**D**) Heart weight/tibial length (HW/TL) ratio was significantly reduced 16 weeks after STZ treatment in both mice. **p < 0.01, ***^,###^p < 0.001.

To study whether reduced FA use influences the cardiac function in the context of diabetes by insulin deficiency, cardiac function was assessed by echocardiography for 16 weeks after STZ treatment. Body weight was significantly reduced by STZ treatment, which was accompanied by disappearance of abdominal and subcutaneous adipose tissue (Supplementary Fig. S1). While functional difference was not observed between DKO and WT mice at baseline, a decrease in fractional shortening (FS) and an increase in left ventricular systolic diameter (LVESD) was more remarkable in DKO-STZ mice at all measured time points after 8 week (Fig. 1A). Wall thickness of interventricular septum (IVS) and posterior wall (PW) was not different among all groups (Supplementary Fig. S2). Systolic blood pressure and heart rate were not significantly different between the groups with and without STZ treatment (Fig. 1B,C). Cardiac atrophy was observed by heart weight/tibial length (HW/TL) ratio and cross sectional area (CSA) in STZ-treated mice (Fig. 1D and Supplementary Fig. S3). These findings suggest that contractile dysfunction in DKO-STZ mice is more prominent compared to that in WT-STZ although cardiac atrophy is induced by STZ in both mice to a similar extent.

### Glucose uptake is not reduced in DKO-STZ hearts

**Figure 2:**
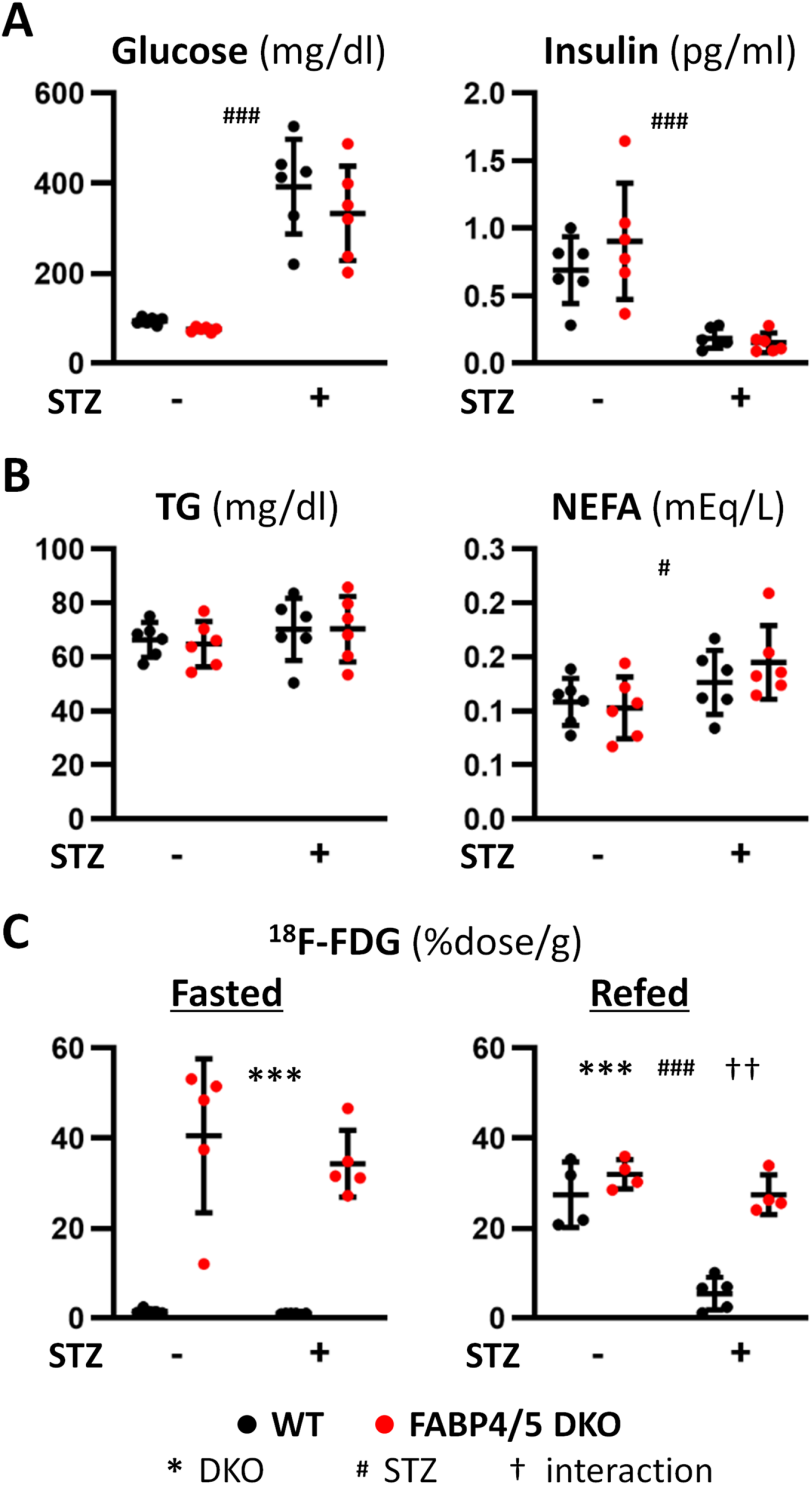
Circulating levels of metabolites and uptake of glucose tracer by hearts in the absence or the presence of STZ treatment. Samples were collected after a 6 h fast (n = 6). (**A**) Serum levels of glucose and insulin. (**B**) Serum levels of TG and NEFA. (**C**) Uptake of ^18^F-FDG, glucose tracer, by hearts. TG, triacylglycerol; NEFA, non-esterified fatty acids. ^#^p < 0.05, ^††^p < 0.01, ***^,###^p < 0.001.

We examined metabolic alteration by STZ treatment. Blood glucose levels were markedly increased by STZ treatment while insulin levels were reduced (Fig. 2A). Serum levels of non-esterified FA (NEFA) were increased by STZ treatment (Fig. 2B). We next examined effects of diabetes on uptake of ^18^F-FDG, glucose tracer. ^18^F-FDG uptake was 20-fold higher in DKO hearts compared to WT at baseline to compensate for reduced FA uptake (Fig. 2C). ^18^F-FDG uptake was not changed by STZ treatment in both groups in the fasted state (Fig. 2C). However, ^18^F-FDG uptake was markedly reduced after refeeding in WT-STZ mice, but not in DKO-STZ mice. These finding suggest that insulin-dependent glucose uptake by the heart is significantly reduced during postprandial period in WT-STZ mice and that glucose uptake is constantly enhanced without an effect of insulin in DKO-STZ.

### Ketolysis is accelerated in DKO hearts irrespective of STZ treatment

**Figure 3:**
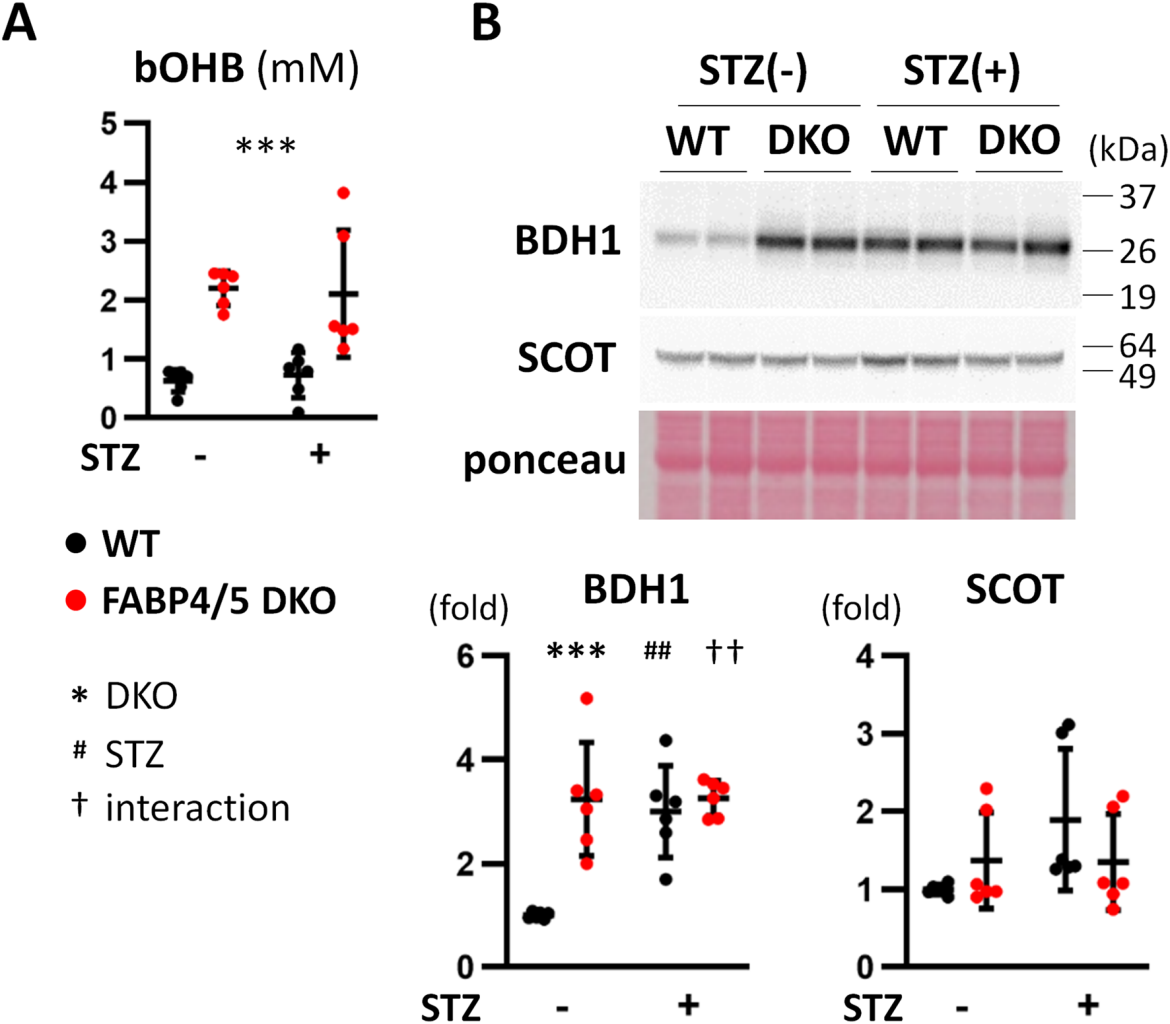
Circulating ketone body and ketolytic enzyme are increased in DKO hearts. (**A**) Serum levels of bOHB, β-hydroxybutyrate (n = 6). (**B**) Protein expression of ketolytic enzymes, BDH1 and SCOT, was assessed by western blot analysis (n = 6). ^##,††^p < 0.01, ***p < 0.001.

Ketone bodies are crucial fuels for hearts in diseased conditions, such as diabetes and heart failure^20^. We found that serum levels of β-hydroxybutyrate (bOHB) were increased in DKO mice after a 6 h fast irrespective of STZ treatment (Fig. 3A). Expression of BDH1, a ketolytic enzyme, was enhanced in DKO-control, WT-STZ and DKO-STZ hearts compared to WT-control whereas that of SCOT, another ketolytic enzyme, was not changed (Fig. 3B and Supplementary Fig. S8). These findings suggest that ketone body utilization seems to be facilitated in DKO hearts to compensate for reduced FA supply irrespective of STZ treatment.

### Facilitated glycolysis in DKO hearts is diminished by STZ treatment

**Figure 4:**
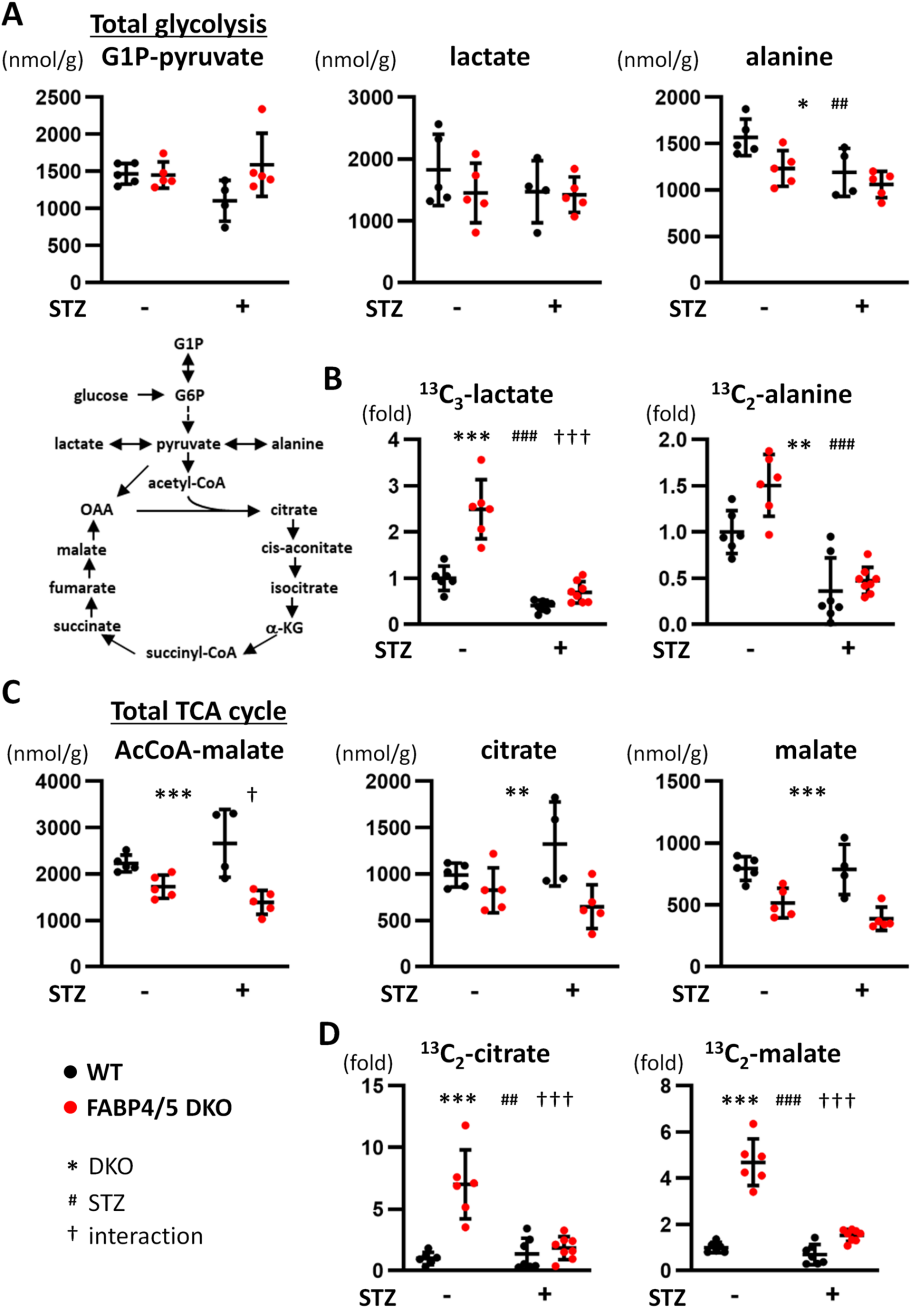
(**A**,**B**) Glycolytic flux was suppressed by STZ treatment. (**A**) Metabolic profiling in glycolysis pathway. G1P-pyruvate, total metabolites from G1P to pyruvate in glycolysis (n = 4–5). Hearts were isolated after a 6 h fast for metabolome analysis. (**B**) Tracer study with ^13^C_6_-glucose. After a 6 h fast, hearts were isolated 10 min after intraperitoneal injection of ^13^C_6_-glucose (n = 6). (**C**,**D**) The pool size in the TCA cycle was significantly reduced in DKO-STZ hearts. (**C**) Metabolic profiling in the TCA cycle. (n = 4–5). Hearts were isolated after a 6 h fast for metabolome analysis. (**D**) Tracer study with ^13^C_6_-glucose. After a 6 h fast, hearts were isolated 10 min after intraperitoneal injection of ^13^C_6_-glucose (n = 6). *G1P* glucose-1-phosphate, *G6P* glucose 6-phosphate, *α-KG* α-ketoglutarate, *OAA* oxaloacetate. *^,†^p < 0.05, **^,##^p < 0.01, ***^,###,†††^p < 0.001.

To examine cellular metabolic changes in DKO hearts, metabolome analysis was carried out. Despite a marked increase in glucose uptake in DKO mice (Fig. 2C), total metabolites in glycolysis (G1P to pyruvate) and levels of lactate were comparable between WT and DKO hearts irrespective of STZ treatment (Fig. 4A). Levels of alanine were lower in DKO hearts and reduced by STZ treatment (Fig. 4A). Tracer study with ^13^C_6_-glucose revealed that levels of ^13^C_3_-lactate and ^13^C_2_-alanine were significantly elevated in DKO hearts compared to WT, and the glycolytic flux was suppressed by STZ treatment (Fig. 4B). These data suggest that accelerated glycolytic rate in DKO hearts is significantly reduced by insulin deficiency despite a fact that enhanced glucose uptake is maintained.

### Accelerated glycolytic flux into the TCA cycle in DKO hearts is suppressed by STZ treatment

We next explored the amounts of metabolites in the TCA cycle. The pool size of total metabolites in the TCA cycle (acetyl-CoA to malate) was significantly lower in DKO hearts at baseline and the difference was enhanced by STZ treatment (Fig. 4C, interaction p < 0.05). Consistent with this, each metabolite in the TCA cycle such as citrate and malate exhibited tendency similar to the pool size (Fig. 4C). These findings suggest that increased FA use in diabetic WT hearts is compensation for reduced glucose utilization, which is lost in DKO-STZ hearts. Contrary to the reduction in the pool size in the TCA cycle, isotopomer analysis with ^13^C_6_-glucose exhibited that levels of ^13^C_2_-citrate and ^13^C_2_-malate were significantly increased in DKO hearts at baseline (Fig. 4D), which strongly suggests that accelerated glycolytic flux is compensation for reduced FA uptake. Although levels of ^13^C_2_-citrate and ^13^C_2_-malate were not markedly changed by-STZ treatment in WT, they were strongly reduced by STZ in DKO hearts (Fig. 4D, interaction p < 0.001). Thus, an accelerated glycolytic flux into the TCA cycle in DKO-STZ hearts is remarkably suppressed despite sustained enhancement of glucose uptake. We also measured levels of adenosine triphosphate (ATP) and phosphocreatine (PCr), reserve energy, to estimate creatine-phosphate energy shuttle. ATP levels were reduced by STZ treatment whereas PCr levels were not changed (Supplementary Fig. S4). There was a decline in ATP levels even in WT-STZ hearts despite maintained cardiac contraction. Because it is known that a decline in PCr levels usually precedes a decrease in ATP^21^, interpretation of early reduction in ATP levels remains elusive. Together, metabolic flux from two major fuels, FA and glucose, into the TCA cycle seems to be more reduced in DKO-STZ hearts, which could account for enhanced reduction in the pool size in the TCA cycle (or reduced energy supply relative to energy expenditure, see the discussion in detail) and a resultant deterioration of contractile dysfunction. Our data also suggest that the pool size in the TCA cycle is likely to be a more promising marker to estimate energy status in the failing heart compared to PCr/ATP.

### Levels of most ceramides are elevated by STZ treatment in WT and DKO hearts

**Figure 5:**
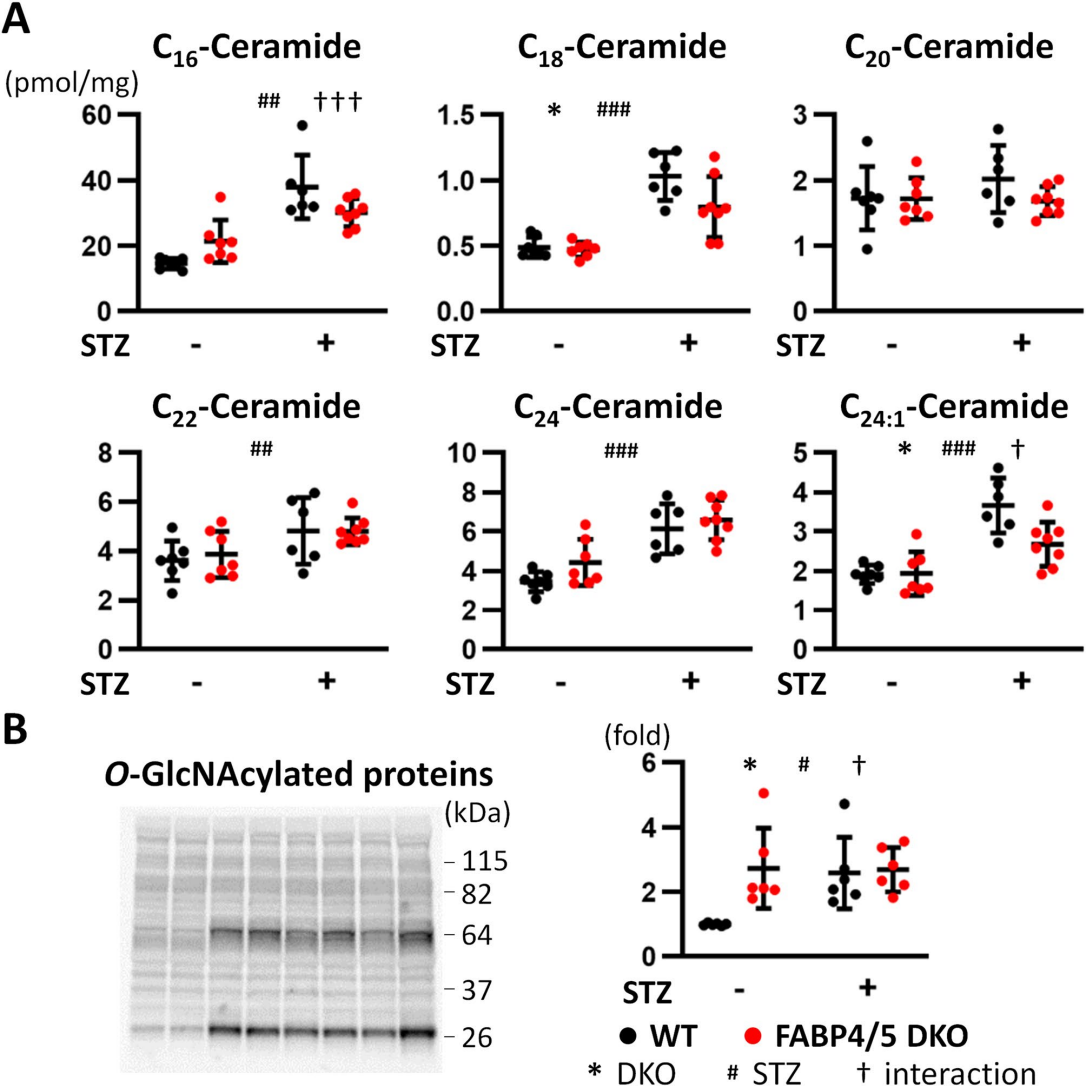
(**A**) Accumulation of cardiotoxic lipids was not enhanced in DKO-STZ hearts compared to WT-STZ. Levels of ceramides with different length of FAs; C_16_, C_18_, C_20_, C_22_, C_24_ and C_24:1_ (n = 6). (**B**) *O*-GlcNAcylation was similarly induced in WT-STZ and DKO-STZ hearts. *O*-GlcNAcylation of extracted proteins was estimated by western blot analysis with anti-GlcNAc antibody (n = 6). *^,†^p < 0.05, ^##^p < 0.01, ^###,†††^p < 0.001.

Diabetes induces an imbalance between FA uptake and oxidation, which leads to accumulation of cardiotoxic lipids such as ceramides and DAG^4–8^. Amounts of most ceramides with different length of FAs were elevated by STZ treatment in both mice compared to control groups (Fig. 5A). However, there was interaction between genotype (DKO) and STZ treatment in C_16_- and C_24:1_-ceramides (Fig. 5A), indicating a significant reduction in DKO-STZ hearts compared to WT-STZ. Importantly, amounts of C_16_- and C_24:1_-ceramides occupy more than half of whole ceramides. Thus, accumulation of lipotoxic lipids does not account for exacerbation of cardiac dysfunction observed in DKO-STZ hearts.

### GlcNAcylation of proteins is similarly induced in WT-STZ and DKO-STZ hearts

Excessive glucose availability has also been explored as a contributing factor to cellular dysfunction in diabetes, which is known as glucotoxicity^5,22,23^. Hexosamine biosynthesis pathway (HBP) is an ancillary pathway of glucose metabolism to modify protein function via *O*-linked attachment of UDP-GlcNAc (referred to as *O*-GlcNAcylation)^22–25^. Given that accelerated glucose uptake was not suppressed by STZ treatment, we presumed that *O*-GlcNAcylation might be a key mechanism for reduced cardiac contraction. Interestingly, GlcNAcylated proteins were already enhanced in cellular extracts from DKO hearts without STZ treatment (Fig. 5B and Supplementary Fig. S2). In addition, similar distribution of GlcNAcylated proteins was observed in WT-STZ and DKO-STZ hearts (Fig. 5B and Supplementary Fig. S9). These findings suggest that *O*-GlcNAcylation is unlikely to be a cause of deteriorated contractile dysfunction observed in DKO-STZ hearts.

### Other possible causes of contractile dysfunction in DKO-STZ hearts

It has been suggested that augmented inflammatory responses and mitochondrial damage are possible mechanisms to facilitate metabolic impairment in diabetic hearts^4,5,7^. Expression of pro-inflammatory cytokines, such as tumor necrosis factor (*Tnf*) and interlukin 1β (*Il1b*), was not induced in DKO-STZ hearts (Supplementary Fig. S5), which suggests that inflammation is not a major factor in etiology of reduced contraction in DKO-STZ hearts. Overt mitochondrial damage, estimated by protein expression of electron transport chain complex, was not observed in DKO-STZ hearts, either (Supplementary Fig. S6). Thus, inflammatory responses and mitochondrial damage are unlikely to be responsible for reduced contractility in DKO-STZ hearts.

Expression of a master regulator for FA catabolism, *Ppara* (peroxisome proliferator activated receptor α), was induced by STZ treatment in both mice (Supplementary Fig. S7). However, expression of other FA metabolism-related genes such as *Cd36*, *Acadm* (acly-CoA dehydrogenase medium chain), *Acadl* (acly-CoA dehydrogenase long chain) and *Fabp3* (heart-type FABP) was lower in DKO-STZ hearts compared to WT-STZ. Reduction of their gene expression might be caused by a decrease in endogenous PPARA ligands due to reduced FA uptake. These findings suggest that transcriptional suppression for FA metabolism could further enhance a reduction in FA utilization in DKO-STZ hearts compared to WT-STZ.

## Discussion

In this study, our results suggest that aggravation of contractile dysfunction in DKO-STZ hearts is due to energy insufficiency. Total energy supply relative to energy expenditure, estimated by the pool size in the TCA cycle, was significantly reduced in DKO-STZ hearts for the following reasons. First, FA uptake by DKO hearts is diminished due to reduced function of trans-endothelial FA transport as reported previously^16^. Second, the enhanced glycolytic rate was markedly suppressed in DKO-STZ hearts. Although compensatory glucose uptake was maintained, glycolytic flux into the TCA cycle was strongly suppressed. An increase in ketone body utilization was also insufficient for restoration of energy shortage. Thus, a reduction in total energy supply is caused by reduced FA uptake and suppressed glycolysis, which could account for exacerbated contractile dysfunction. We further suggest that in the aspect of energy homeostasis, enhanced FA uptake in diabetic hearts seems to be a compensatory response to reduced energy supply from glucose, and therefore, limited FA use could contribute to deterioration of cardiac contractile dysfunction in diabetes, rather than protection from lipotoxic effects.

Our study also addressed other possibilities of cardiac contractile dysfunction in diabetes in addition to energy shortage. We showed no increase in accumulation of cardiotoxic lipids such as ceramides in DKO-STZ hearts compared to WT-STZ. Further, *O*-GlcNAcylation was similarly induced in WT-STZ and DKO-STZ hearts. We also observed no obvious inflammatory responses and mitochondrial damage. These findings suggest that lipotoxicity, glucotoxicity, inflammatory responses and mitochondrial damage are not major reasons for enhanced contractile dysfunction in DKO-STZ hearts.

### The pool size in the TCA cycle as a useful marker for energy status in vivo beating hearts

**Figure 6:**
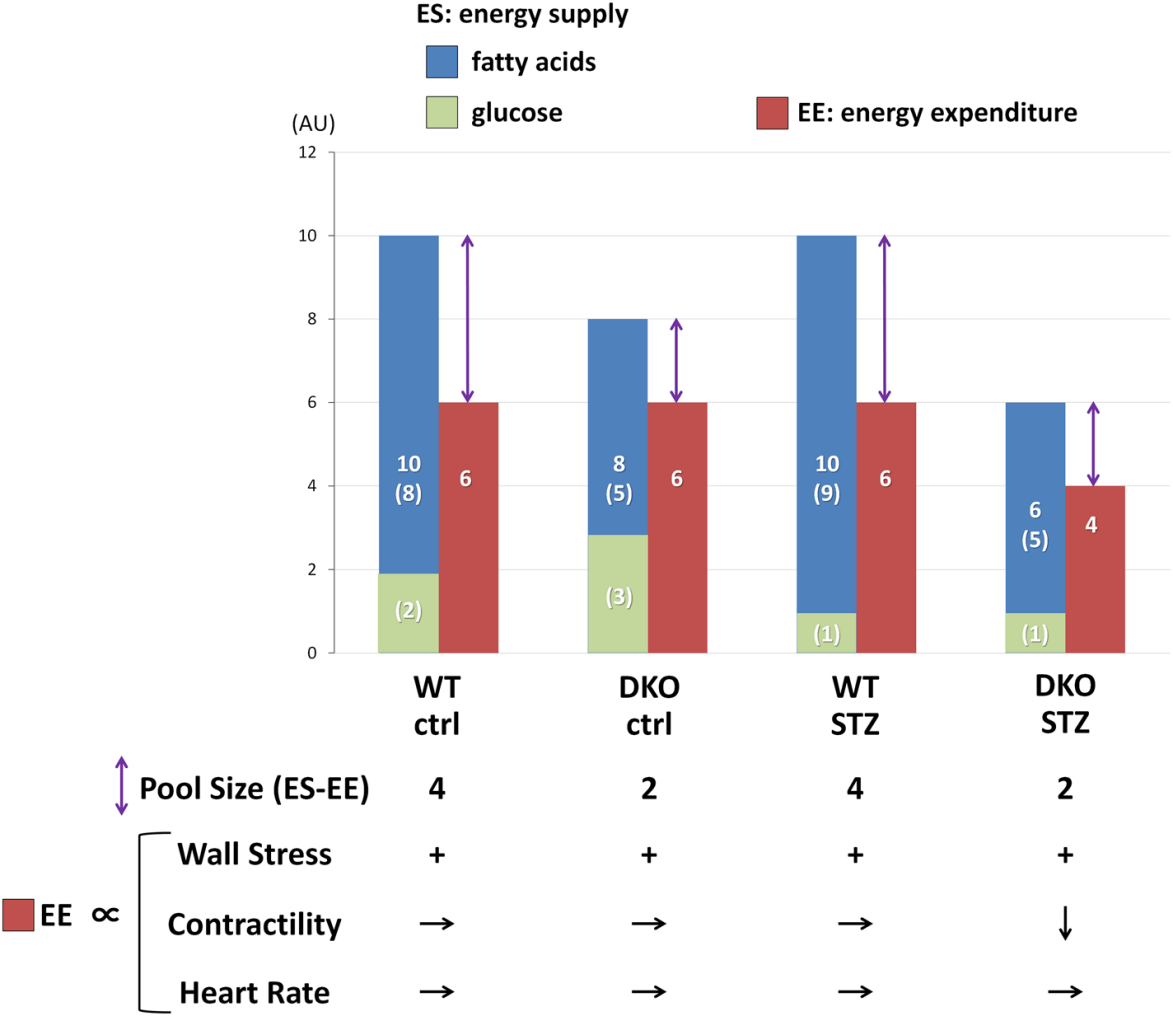
Putative bar graph regarding the pool size in the TCA cycle. There are two points to understand the working hypothesis. First, the pool size in the TCA cycle is associated with balance between energy supply (ES) and energy expenditure (EE). Second, EE is determined by three components, wall stress, contractility and heart rate (see “Discussion” in detail). *AU* arbitrary unit.

The pool size in the TCA cycle has been shown as a marker for energy status in isolated working hearts^26–30^. Keeping a constant amount of the pool size is necessary for normal function of energy production and cardiac contraction. Recently, we proposed that the pool size is also a useful marker for energy balance in vivo beating hearts under increased workload by transverse aortic constriction [38, 48]. As shown in Fig. 6, the concept of the pool size is also applied to account for our diabetic heart model. We suggest that the pool size is determined by balance between energy supply (ES) and energy expenditure (EE). ES depends on uptake and oxidation of energy substrates, mainly FA and glucose, and lesser amount of ketone bodies. EE is determined by three components, wall stress, contractility and heart rate. In DKO hearts at baseline, ES is smaller compared to WT-ctrl hearts, leading to the reduced pool size in the TCA cycle. The reduced ES in DKO hearts is sufficient for basal cardiac contraction since the reduced ES is still able to fulfill minimal EE required for normal function. In WT-STZ-hearts, FA utilization is enhanced to compensate for suppressed glycolysis, which can maintain total ES and cardiac contraction. In DKO-STZ hearts, however, FA uptake is limited in addition to suppressed glycolysis, which leads to a reduction in total ES. Importantly, cardiac contraction, a determinant for EE, is reduced in DKO-STZ hearts, which implies that the EE is smaller than minimal EE required for normal function. The pool size in DKO-STZ hearts is similar or reduced a little compared to DKO-control (Fig. 6), which further supports the notion that both ES and EE are reduced in DKO-STZ hearts. Thus, a reduction in the pool size in combination with a decrease in cardiac contraction reflects that the ES in DKO-STZ hearts is energetically insufficient for normal cardiac function.

### Significance of lipotoxicity in diabetic cardiomyopathy

The highlight of this study is to show that facilitated FA utilization in diabetic hearts is necessary to keep total energy supply for normal cardiac function. This is the first report to demonstrate that accelerated FA use is beneficial rather than detrimental, which is a sharp contrast to a hitherto known concept of lipotoxicity in diabetic hearts. We presume that the discrepancy occurs because of distinct experimental conditions (ex vivo vs. in vivo) as described below.

In addition to accumulation of cardiotoxic lipids such as ceramides and DAG, it is also suggested that lipotoxic effects appear as a result of inefficient oxygen consumption (MVO_2_)^4,5,7^. While increased FA oxidation is associated with increased MVO_2_, increased MVO_2_ is not accompanied by an equivalent increase in cardiac contractility, which causes a reduction in cardiac efficiency (cardiac work/MVO_2_). Therefore, the reduced cardiac efficiency by excessive FA consumption has been considered to be one of most essential pathophysiological causes in diabetic hearts. However, the concept is established mostly by using isolated working hearts^5,6^. Caveats of such ex vivo studies are that experiments are performed under the same perfusate conditions (e.g. the same concentration of glucose, palmitate, insulin, Ca^2+^, etc.). In diabetic hearts in vivo, however, circulating components are considerably different from those in control hearts. If diabetic hearts are adapted to worse metabolic conditions to exert the best hemodynamic performance in vivo, the beneficial aspect of metabolic adaption is underestimated ex vivo under the same conditions. In this study, we exhibited for the first time that increased FA use in diabetic hearts is energetically compensatory in vivo, something which cannot be determined by ex vivo experiments. Future studies are needed to establish new methods to evaluate effects of metabolic adaptation in diabetic hearts in vivo.

### Significance of glucotoxicity in diabetic cardiomyopathy

Because DKO-control hearts with normal cardiac contraction displays enhanced *O*-GlcNAcylation of proteins comparable with those of DKO-STZ, it is unlikely that *O*-GlcNAcylation alone causes reduced cardiac contractility in DKO-STZ hearts. We sought for the reason why our data are inconsistent with prevailing idea. *O*-GlcNAcylation is facilitated via the HBP depending on glucose availability in cells^31,32^ and ten to twenty % of all proteins are thought to be possible targets of *O*-GlcNAcylation. *O*-GlcNAcylation is controlled by a pair of enzymes, *O*-GlcNAc transferase (OGT) and *O*-GlcNAcase (OGA) and the modification cycle is very rapid, which takes 1 min or even less^31–33^. Thus, the HBP has wide range of target proteins, which is rapidly, reversibly and non-selectively controlled by a pair of enzymes and transient nutritional state. Based on such characteristics of *O*-GlcNAc modification, we presume that comparable levels of *O*-GlcNAcylation should have similar effects on functions of target proteins. This notion is supported by our data that accelerated *O*-GlcNAcylation alone did not cause reduced cardiac contractile function in DKO-control hearts and that WT-STZ hearts did not exhibit contractile dysfunction at earlier phase of diabetes. Although it is proposed that *O*-GlcNAcylation of several candidate proteins can cause cardiac dysfunction^22–25,34^, our findings raise another possibility that some additional mechanisms exist to exacerbate protein function modified by *O*-GlcNAcylation in diabetic hearts during later period of diabetes.

## Conclusions

Increased FA use in diabetic hearts is required for preservation of cardiac contraction to compensate for reduced energy supply from glucose. Therefore, limited FA utilization could be detrimental to cardiac contractile dysfunction in diabetic hearts, rather than protective against lipotoxicity.

## Methods

### Mice, type I diabetic model and sample preparation

All studies were approved by the Institutional Animal Care and Use Committee (Gunma University Graduate School of Medicine). Animal experiments were carried out according to the NIH guidelines (Guide for the Care and Use of Laboratory Animals). FABP4 and FABP5 double knockout (DKO) mice with the C57BL6j background were generated as described previously^35^. Control wild type mice (WT) and DKO^−/−^ were obtained by breeding DKO^+/−^ (*Fabp4*^+*/*−^*Fabp5*^+*/*−^) male and female mice. The mice were housed in a temperature-controlled room (20–26 °C) with a 12-h light/12-h dark cycle and given unrestricted access to water and standard chow (CE-2, Clea Japan, Inc.). For the type 1 diabetic model, 10–12-week-old male mice were treated with i.p. STZ in 0.1 M sodium citrate (pH 4.5) at a dose of 50 mg/kg for 5 days^36^. Blood was collected from the retro-orbital plexus, and then centrifuged at 1500 × *g* for 15 min at 4 °C to obtain the serum for measurement of biochemical parameters. After cervical dislocation, hearts were dissected, snap frozen in liquid nitrogen and stored at − 80 °C until further use.

### Cardiac function and hemodynamic parameters

The in vivo cardiac function and the heart rate were assessed by transthoracic echocardiography (EUB-7500, Hitachi, Tokyo) in conscious mice as described previously^19,37^. Blood pressure data were obtained from the average of three-time measurements by the tail cuff method (MK-2000ST, Muromachi Kikai, Tokyo)^18^.

### Measurement of blood metabolites

Serum levels of glucose (glutest sensor, Sanwa Kagaku, Aichi), triglyceride (Triglyceride E-test, Wako Chemical, Osaka), non-esterified fatty acid (NEFA C-test Wako Chemical, Osaka) and β-hydroxybutyrate (EnzyChrom Ketone Body Assay Kit, BioAssay Systems, California) and insulin (mouse Insulin ELISA, Mercodia, Sweden) were measured according to the manufacturer’s protocols^18,19,37^.

### The biodistribution of ^18^F-FDG (2-Fluorodeoxyglucose)

Mice received intravenous injections of ^18^F-FDG (100 kBq) via the lateral tail vein in a volume of 100 μl. ^18^F-FDG was obtained from batches that were prepared for clinical PET imaging at Gunma University. The animals were sacrificed 2 h after injection. The isolated hearts were weighed and counted using a well-type gamma counter (ARC-7001, Hitachi-Aloka Medical)^18,19,37^.

### Western blot analysis

Western blot analysis was done with antibodies against 3-hydroxybutyrate dehydrogenase 1 (BDH1, SCB, sc-134281, CA) and succinyl CoA:3-oxoacid CoA transferase (SCOT, SCB, sc-133988, CA) as described previously^18,19,37^. To avoid removal of GlcNAcylation of proteins, proteins were extracted with the RIPA buffer containing an *O*-GlcNAcase inhibitor, *O*-(2-acetamido-2-deoxy-d-glucopyranosylidenamino) *N*-phenylacarbamate (i.e. PUGNAc, 25 μM)^38^. GlcNAc modification was detected with *O*-GlcNAc antibody (CTD110.6, SCB, sc-59623, CA).

### Metabolome analysis by capillary electrophoresis-mass spectrometry

The mice were anesthetized by isoflurane and the ventricles were immediately removed from the mice after a 6 h fast. The heart samples were freeze-clamped using aluminum blocks precooled in liquid nitrogen and maintained at – 80 ℃. Metabolome analyses were carried out as described elsewhere^16,18,19,37^.

### Tracing study with ^13^C_6_-glucose

Six hours after fasting, ^13^C_6_-glucose (1 mg/g) was intraperitoneally injected into mice. Ten minutes later, the ventricles were isolated, and metabolome analyses were conducted as described previously^18,19,37^.

### Measurement of ceramides

Ceramide measurement was performed by using a triple quadrupole mass spectrometer coupled with a liquid chromatograph (LCMS-8050 system, Shimadzu). Heart tissues were crashed to powder in liquid nitrogen, then rotated for 1 h at 4 °C in methanol containing 1 μM C18 ceramide (C17 base, d17:1/18:0) as an internal standard. The extract was separated on a reversed-phase C8 column (Kinetex C8, 2.1 mm × 150 mm, 2.6 μm, Phenomenex) by using a gradient of solvent A (water/methanol/formic acid 97/2/1) and B (methanol/acetone/water/formic acid 68/29/2/1) with a flow rate of 0.4 ml/min. The initial solvent composition was 60% B, and the following solvent gradient was applied: 60% for 1 min, increased linearly to 100% from 1 to 8 min, held at 100% B for 3 min, then returned to 60% B and maintained for 2 min. The column was maintained at 60 °C. The separated analytes were ionized by electrospray ionization, then measured by the mass spectrometer with selected reaction monitoring (SRM) mode. The SRM transition for analytes were *m/z* 538.5 > 264.2 [M + H]^+^ for C16 ceramide (d18:1/16:0), *m/z* 548.5 > 264.2 [M + H-H_2_O]^+^ for C18 ceramide (d18:1/18:0), *m/z* 576.5 > 264.2 [M + H-H_2_O]^+^ for C20 ceramide (d18:1/20:0), *m/z* 604.5 > 264.2 [M + H-H_2_O]^+^ for C22 ceramide (d18:1/22:0), *m/z* 630.5 > 264.2 [M + H-H_2_O]^+^ for C24:1 ceramide (d18:1/24:1), *m/z* 632.5.5 > 264.2 [M + H-H_2_O]^+^ for C24 ceramide (d18:1/24:0), and *m/z* 534.5 > 250.2 [M + H-H_2_O]^+^ for C18 ceramide (C17 base, d17:1/18:0). Peak area of each ceramide was divided by that of the internal standard, then applied to the standard curve made for each ceramide species for quantification. All of the ceramide standards were purchased from Avanti Polar Lipids.

### Statistical analysis

Statistical analysis was performed with IBM SPSS (version 24, IBM, NY). The data are presented as mean ± standard deviation. Student’s t-test was performed for 2 groups’ comparison. Two-way analysis of variance (ANOVA) was used to analyze effects of genotype (WT vs. DKO), STZ treatment (control vs. STZ) and their interaction. A p value < 0.05 was considered to be statistically significant. *p < 0.05, **p < 0.01, ***p < 0.001; main effect for genotype. ^#^p < 0.05, ^##^p < 0.01, ^###^p < 0.001; main effect for STZ treatment. ^†^p < 0.05, ^††^p < 0.01, ^†††^p < 0.001; interaction between genotype and STZ treatment.

### Ethics approval and consent to participate

All studies were approved by the Institutional Animal Care and Use Committee (Gunma University Graduate School of Medicine, 18-080).

### Consent for publication

All authors have declared their consent for this publication.

## Supplementary information

Supplementary Information.

## Data Availability

All data and materials are available upon request.
