# Supplementary material for: Reduced fatty acid uptake aggravates cardiac contractile dysfunction in streptozotocin-induced diabetic cardiomyopathy
Source: Sci Rep. 2020 Nov 30;10:20809. doi: 10.1038/s41598-020-77895-1 (PMC7705707; doi:10.1038/s41598-020-77895-1)
Supplement: Supplementary file 1 — Supplementary Information. [file 41598_2020_77895_MOESM1_ESM.pdf]

Supplementary information file

Concerning the manuscript with the title
‘Reduced fatty acid uptake aggravates cardiac contractile dysfunction in streptozotocin-induced diabetic cardiomyopathy’

Authors
Yogi Umbarawan, Ryo Kawakami,
Mas Rizky A.A Syamsunarno, Norimichi Koitabashi, Hideru Obinata, Aiko Yamaguchi, Hirofumi Hanaoka, Takako Hishiki, Noriyo Hayakawa, Hiroaki Sunaga, Hiroki Matsui, Masahiko Kurabayashi, Tatsuya Iso

Supplemental Methods

Cross sectional area

Hearts were fixed with 10% formalin, embedded in paraffin and sectioned into 8 µm slices. To assess cardiomyocyte cross-sectional area, tissue was stained with wheat germ agglutinin (WGA; plasma membrane staining, Alexa Fluor 488 conjugated).

RNA isolation and real time PCR

Total RNA was isolated from hearts using the TRIzol Reagent (Invitrogen). Quantitative real-time PCR was performed with SYBR Green PCR Master Mix (Applied Biosystems), according to the manufacturer's instructions. The expression level of the target gene was normalized to the GAPDH (glyceraldehyde-3-phosphate dehydrogenase) mRNA level. The ready-to-use gene-specific primers for cDNA (Perfect Real Time Primer) were purchased from Takara Bio Inc. (Shiga, Japan).

Supplementary figure 1

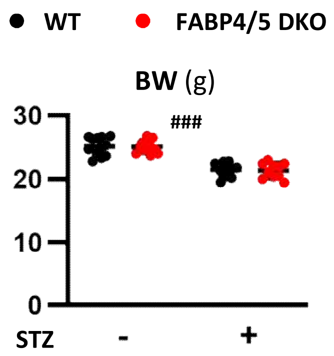

Figure S1. Body weight (BW) was significantly reduced by STZ treatment (n=10-12). STZ ###$p<0.001$.

Supplementary figure 2

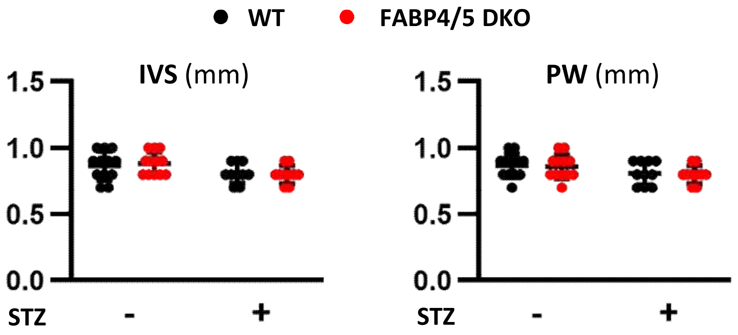

Figure S2. Wall thickness of left ventricles estimated by echocardiography (n=10-12). IVS; interventricular septum, PW; posterior wall.

Supplementary figure 3

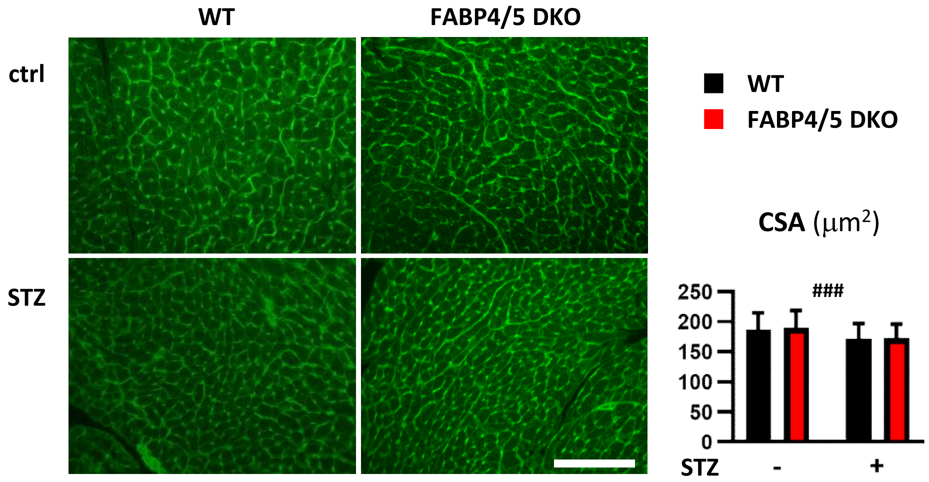

Figure S3. Cross sectional area (CSA) was reduced by STZ treatment in both mice (n=6). Scale bar = 100 mm. STZ $^{###}p<0.001$.

Supplementary figure 4

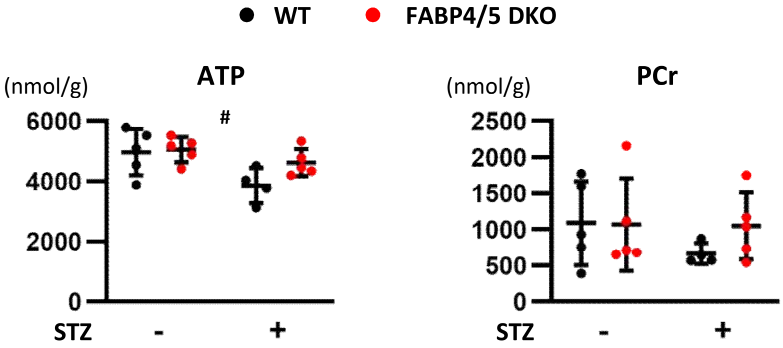

Figure S4. ATP and PCr levels measured by metabolome analysis (n=4-5). ATP; adenosine triphosphate, PCr; creatinine phosphate. STZ #p<0.05.

Supplementary figure 5

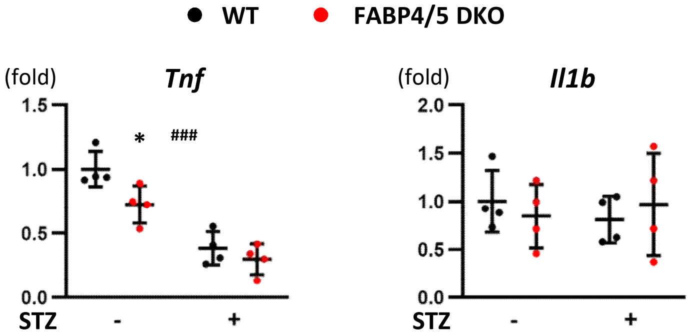

Figure S5. Expression levels of mRNA for *Tnf* and *Il1b* (n=4). Tnf: tumor necrosis factor α, Il1b; interleukin-1β. DKO *p<0.05, STZ ###p<0.001.

Supplementary figure 6

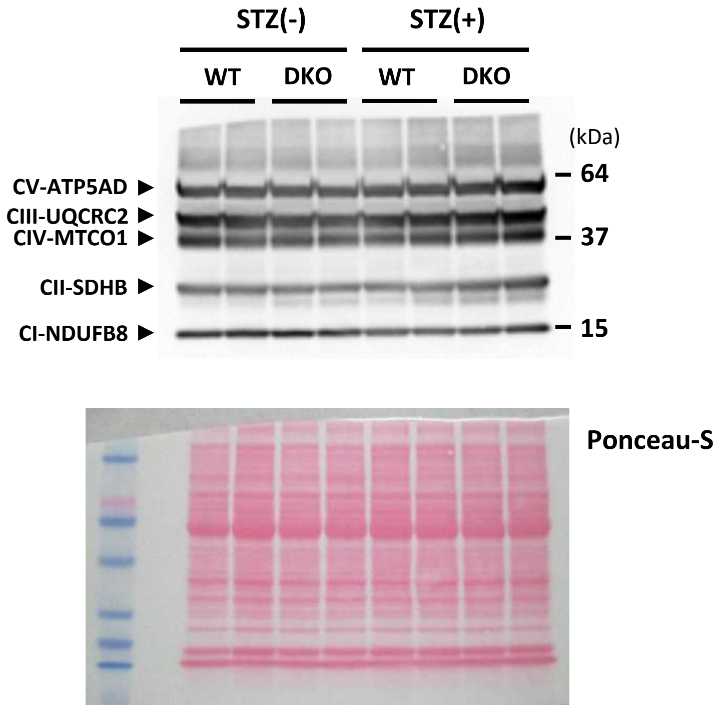

Figure S6. Full length western blots of representative proteins in mitochondrial electron transport chain complex. Equal amount of loaded protein was confirmed by ponceau-S staining. The 1st antibody used was total OXPHOS rodent western blot antibody cocktail (Abcam110413, MA). CV-ATP5A; complex V-ATP synthase subunit alpha, CIII-UQCRC2; complex III-cytochrome b-c1 complex subunit 2, CIV-MTCO1; complex IV-cytochrome c oxidase subunit 1, CII-SDHB; complex II-succinate dehydrogenase subunit B, CI-NDUFB8; complex I- NADH dehydrogenase (ubiquinpone) 1 beta subunit 8.

Supplementary figure 7

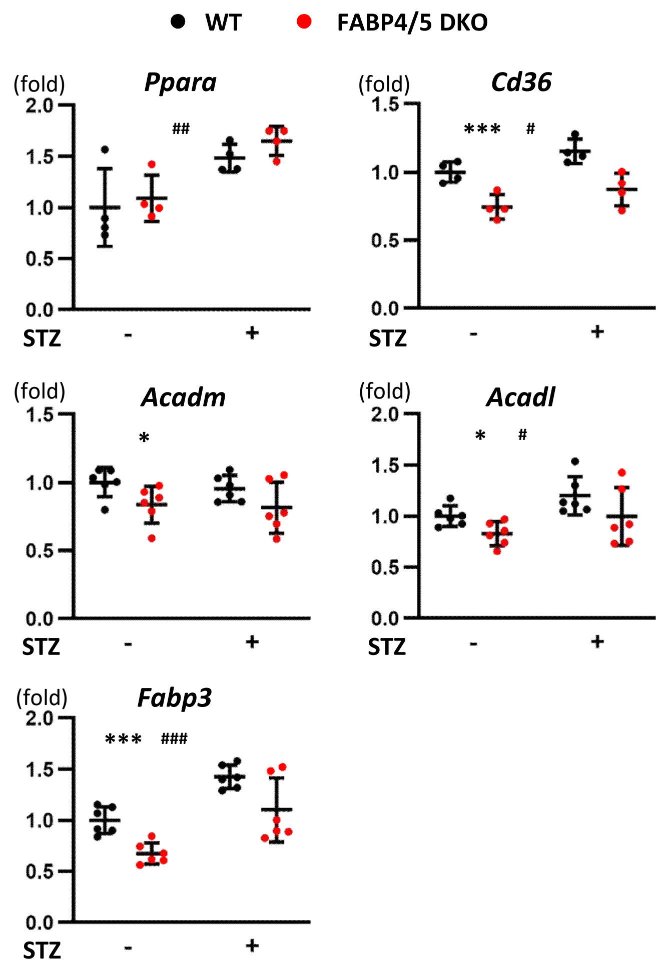

Figure S7. Expression levels of mRNA for *Ppara, Cd36, Acadm, Acadl* and *Fabp3* (n=6). Ppara: peroxisome proliferator activated receptor α, Acadm; acyl-CoA dehydrogenase medium chain, Acadl; acyl-CoA dehydrogenase long chain, Fabp3; fatty acid binding protein 3.
DKO $^{*}p<0.05$, $^{***}p<0.001$,
STZ $^{\#}p<0.05$, $^{\#\#}p<0.01$, $^{\#\#\#}p<0.001$.

Supplementary figure 8

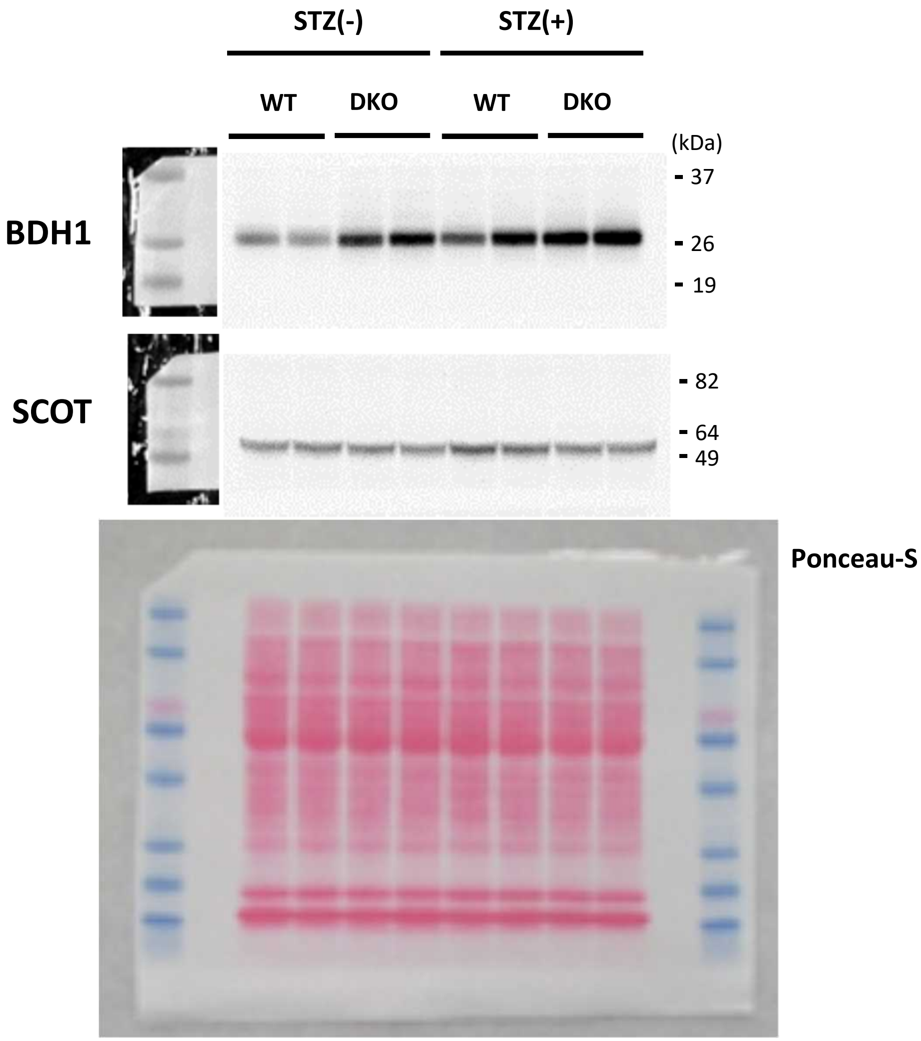

Figure S8. Full length western blots of figure 3B.
Membranes were cut after ponceau-S staining to save 1st antibodies.

Supplementary figure 9

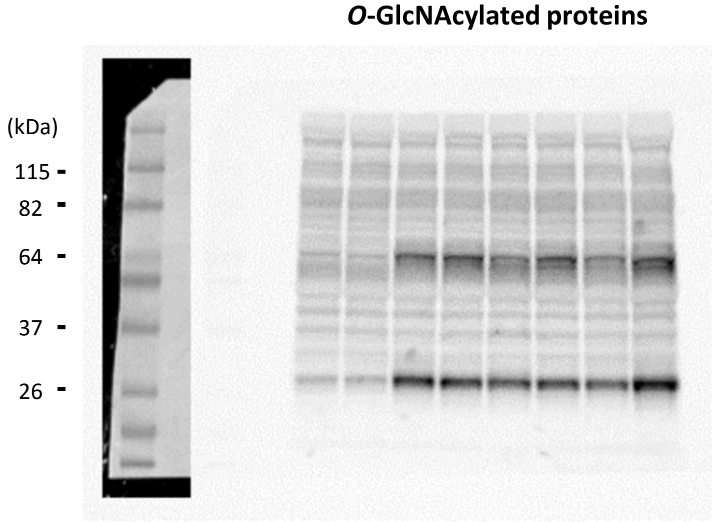

Figure S9. Full length western blot of figure 5B.
